# NADPH oxidases as electrochemical generators to produce ion fluxes and turgor in fungi, plants and humans

**DOI:** 10.1098/rsob.160028

**Published:** 2016-05-18

**Authors:** Anthony W. Segal

**Affiliations:** Division of Medicine, UCL, 5 University Street, London WC1E 6JJ, UK

**Keywords:** fungi, plants, blood pressure, ion channel, membrane

## Abstract

The NOXs are a family of flavocytochromes whose basic structure has been largely conserved from algae to man. This is a very simple system. NADPH is generally available, in plants it is a direct product of photosynthesis, and oxygen is a largely ubiquitous electron acceptor, and the electron-transporting core of an FAD and two haems is the minimal required to pass electrons across the plasma membrane. These NOXs have been shown to be essential for diverse functions throughout the biological world and, lacking a clear mechanism of action, their effects have generally been attributed to free radical reactions. Investigation into the function of neutrophil leucocytes has demonstrated that electron transport through the prototype NOX2 is accompanied by the generation of a charge across the membrane that provides the driving force propelling protons and other ions across the plasma membrane. The contention is that the primary function of the NOXs is to supply the driving force to transport ions, the nature of which will depend upon the composition and characteristics of the local ion channels, to undertake a host of diverse functions. These include the generation of turgor in fungi and plants for the growth of filaments and invasion by appressoria in the former, and extension of pollen tubes and root hairs, and stomatal closure, in the latter. In neutrophils, they elevate the pH in the phagocytic vacuole coupled to other ion fluxes. In endothelial cells of blood vessels, they could alter luminal volume to regulate blood pressure and tissue perfusion.

## 1. Introduction

### 1.1. NOX2 of the neutrophil microbicidal oxidase—the prototype NOX

In 1978, the first NOX was described [1] in human neutrophils. These are the most numerous of the white blood cells, with prime responsibility for killing bacteria and fungi which they engulf into an inverted sac of plasma membrane called the phagocytic vacuole. The phagocytosis of the organisms is associated with a burst of non-mitochondrial respiration [2], called the respiratory burst, that generates 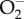 [3] and is important for the efficient killing of the microbes. When this process is defective, it leads to the profound immunodeficiency syndrome of chronic granulomatous disease (CGD) [4].

It transpired that the neutrophil oxidase consists of a flavocytochrome *b*, gp91^phox^, located in the membrane of the vacuole, which requires a number of accessory proteins to enable electron transport from NADPH [5] in the cytosol to O_2_ in the vacuole [6]. Loss, or defective function, of gp91^phox^ [7], or of one of these accessory proteins [8–10] leads to failure of the oxidase, resulting in CGD.

### 1.2. The NOX2 electron-transporting cassette

gp91^phox^ is a short electron transport chain that is perfectly adapted to transfer electrons across a membrane. The structure has not been derived directly but largely through homology with functionally similar molecules. There are six transmembrane α-helices linked by three external and two cytosol-facing loops and a long cytosolic tail. It had been shown that gp91^phox^ itself contained the binding sites for NADPH and FAD [11]. The cytosolic C-terminus has strong homology to ferredoxin NADP reductase that was used as a template to model the NADPH and FAD binding sites in this region [12]. We showed FRE1 ferric reductase of *Saccharomyces cerevisiae* to be a cytochrome *b* similar to gp91^phox^ [13], and were able to define the haem binding sites on the transmembrane helices by mutating the relevant histidines, which placed the haems about 12 amino acids apart, near either surface of the membrane [14]. The location of carbohydrate binding sites helped identify structures located on the external surface of the membrane [15].

There is a second membrane component, p22^phox^, attached to gp91^phox^ [16,17] with which it is in stoichiometric equivalence [18], and both proteins are missing as a result of a damaging mutation in either [19], indicating their mutual interdependence for stability. In neutrophils, the cytoplasmic tail of p22^phox^ acts as a docking mechanism for phosphorylated cytoplasmic p47^phox^ [20].

### 1.3. Other NOXs and homologues exist in animals, plants and fungi

**Figure 1.**
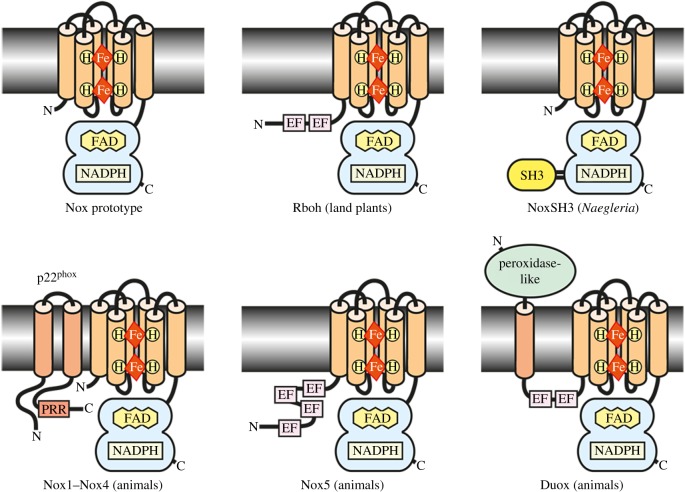
Models for structures of various subtypes of NOX-family enzymes. From [26]. These molecules have a common structure with six transmembrane helices within which are two haems, close to either surface of the membrane. The C-terminal cytosolic tail contains the NADPH and FAD binding sites. Cylinders represent six transmembrane α-helices. EF—Ca^2+^-binding EF-hand motif. PRR—proline-rich region, Src homology-3 domain. DUOX1 and DUOX2 were called dual oxidases because they have an additional ‘peroxidase’-like motif at their N-terminus on the outer surface of the membrane [27]. The product of the DUOXs is H_2_O_2_ rather than the 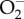 produced by most other NOXs, indicating that these peroxidase domains are in fact acting as superoxide dismutases. Myeloperoxidase can serve the same function in neutrophils [28].

The proliferation of genome sequencing gave rise to the realization that several homologous NOXs existed in man, and then soon after they were also described in other forms of life as diverse as plants and fungi [21–24] which predated them on land [25], suggesting that a common ancestor of the NOX genes emerged at an early stage in the evolution of eukaryotes [26]. The fundamental structure of the electron-transporting cassette initially described in gp91^phox^ is conserved in all these molecules. Greater variation is observed in the mechanisms involved in activating electron transport (figure 1).

Homologues of NOX-family proteins also exist in plants where they are referred to as respiratory burst oxidase homologues (Rboh). The plant oxidases all share FAD and NADPH binding sites, six membrane-spanning domains and calcium-binding EF-hand motifs. This organization most closely resembles the mammalian NOX5 subfamily; no ancestral type of DUOX isoforms has been found in plants.

Fungi contain two NOXs, A and B [29]. Their electron-transporting cassette is homologous to gp91^phox^, and NoxD is the fungal equivalent of p22^phox^ [30].

### 1.4. Activating mechanisms

Electron transport can be induced by adding NADPH to the purified, relipidated, mammalian NOX2, which indicates that this molecule comprises the complete electron-transporting apparatus of the 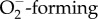 NADPH oxidase [31]. The supplementary molecules required by the diverse systems must activate electron transport by inducing conformational changes in the flavocytochrome.

**Figure 2.**
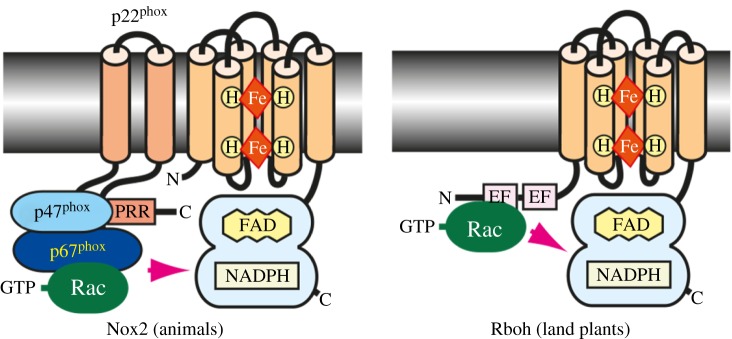
Activating mechanisms in NOXs from animals and plants. From [26]. The activation mechanisms of NOXs 1 and 3 are very similar, with minor variations on this theme [36]. The assembly of the activation complex involves phosphorylation of several of the protein components [36–38] by a variety of kinases and changes to the lipid composition of the membrane by PI3 kinases, and activation of GTP–GDP exchange factors [39]. In addition to activation by binding Ca^2+^, Rbohs can be activated by phosphorylation [40–42].

These activation mechanisms are of two main types. The first is the recruitment of activating proteins to interact with the electron-transporting cassette. In mammalian cells, activation involves movement of proteins from the cytosol to the membranes where they interact with gp91^phox^ and p22^phox^ [32]. These activating proteins include p67^phox^ [33] which is present in the cytosol complexed to p40^phox^ from which it dissociates [33], an interaction that is promoted by the Rho GTPase Rac [34], that must itself dissociate from an inactivating protein GDI [35] and p47^phox^ (figure 2).

NOX5 and DUOX1 and 2 have additional EF-hand motifs at the N-terminus, suggesting their regulation by Ca^2+^ binding [43]. In addition, calcium-dependent kinases, such as protein kinase C and/or Ca^2+^/calmodulin-dependent protein kinase II, are known to phosphorylate and activate NOX5 and DUOXs. DUOXs do not use such complex activation networks, but have two EF-hand calcium binding motifs in the N-terminal cytosolic domain [26] and are activated by the influx of Ca^2+^ [44].

A fungal homologue of p47^phox^ has not been identified, but homologues of the cytosolic p67^phox^ (NoxR) and Rac have been identified in fungi and the scaffold protein Bem1 might be a fungal analogue of p40^phox^.

In contrast to many of the mammalian NOX proteins, regulation of the Rboh proteins is primarily effected through post-translational modifications, such as conformational changes induced by Ca^2+^ binding to their EF-hand motifs [43] and by phosphorylation by Ca^2+^-dependent protein kinases [40,41,45], receptor-like cytoplasmic kinases and mitogen-activated protein kinases [42].

## 2. NOXs as signalling molecules

It is generally believed that NOXs play an important role in ‘signalling’ in fungi [46], plants [47] and mammalian cells [48,49]. The concept of a signalling role for these molecules has arisen because they generate reduced oxygen species (ROS) such as 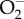 and/or H_2_O_2._ When the NOXs are activated and ROS are generated, a biological effect is observed. When the NOX is missing or inhibited and the ROS are not generated, the biological effect is seen to be absent. The biological effect is then attributed to a direct influence of the ROS, rather than to some other influence of the NOX. As I will detail below, electron transport by NOXs, across the plasma membrane of cells, has dramatic effects upon the pH on either side of the membrane, and on electrochemical-driven ion fluxes into and out of the cell, that themselves influence cellular function without the need to implicate ROS or free radical reactions. In addition, inhibitors of NOXs, such as diphenylene iodonium (DPI) [50], are used in cellular systems to provide evidence that the NOXs are exerting a specified influence on cell function. It is often not appreciated that these inhibitors are not specific. DPI is a general flavoprotein inhibitor and disrupts mitochondrial function [51], with all the subsequent effects on cellular metabolism.

### 2.1. Many mechanisms exist to destroy reactive oxygen species

NOXs transfer electrons across the plasma membrane to O_2_ outside the cell. The 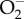 will then be exposed to the extracellular medium, the composition of which is very variable depending upon the specific NOX involved. In root hairs, it will be the soil water solution, in endothelial cells, the blood plasma which has strong antioxidant properties [52] and in fungi, the growth medium. In these situations, 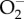 will diffuse rapidly away from the site of generation, and the chances of dismutation to form H_2_O_2_, which is concentration dependent, will rapidly diminish.
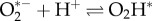


The protonation/deprotonation equilibrium exhibits a p*K*_a_ of 4.88, so that under most circumstances the 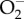 is charged and thereby inhibited from passing across membranes.

The surviving H_2_O_2_ and 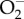 are exposed to the catalase and superoxide dismutase effects of the elements and compounds in the extracellular medium [52] and react with the contained organic materials.

Vitamin E, a potent peroxyl radical scavenger, is a chain-breaking antioxidant that prevents the propagation of free radicals in membranes and in plasma lipoproteins [53]. It is regenerated by vitamin C in the cytosol, itself a potent antioxidant [54], by acting as a soluble reducing agent. Cells living in an oxygen-rich environment have a wide array of antioxidants [55] that remove oxidants such as 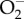 and H_2_O_2_ that penetrate into the cytosol. Cytosolic superoxide dismutase converts 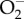 to H_2_O_2_ and catalase breaks down H_2_O_2_ to H_2_O and O_2_. Glutathione and glutathione peroxidase add another arm of reducing power to react with these oxidants [56]. When oxidized, glutathione and ascorbic acid are regenerated from NADPH which is produced in plants by photosynthesis [57] and under other circumstances from glucose by the hexose monophosphate shunt [58].

In addition, to prevent the production of the highly reactive OH^−^ radical by the reaction of 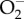 and H_2_O_2_ in the presence of metals such as Cu and Fe, metal binding proteins chelate-free metal [59].

These factors make it very unlikely that free radicals generated extracellularly will be able to function as a reliable and scalable messaging system.

## 3. The biology of the neutrophil phagocytic vacuole as an exemplar of NOX function as an electrochemical pump

The neutrophil was the first biological system in which superoxide generation was recognized as an important biological process [3]. It was initially thought that 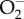 itself, its dismutation product H_2_O_2_, and OH^−^ produced by the interaction of these products catalysed by Fe^2+^ or Cu^2+^ were themselves directly microbicidal, but it has subsequently been recognized that O_2_^−^ and H_2_O_2_ are not reactive enough to kill microbes [60] and free iron and copper are chelated in the vacuole by lactoferrin [59]. So, how does the NOX2 system promote the killing of ingested microbes?

The neutrophil granules make up about 10% of the total cellular protein, which they release into the vacuole containing the ingested microbe. Among their contents are a wide variety of digestive enzymes, the most abundant of which are cathepsin G and elastase, and myeloperoxidase (MPO). There are two main theories as to how the NOX2 oxidase promotes microbial killing, which will not be dealt with in depth here. The dogma has been that it generates H_2_O_2_ as substrate for MPO to oxidize Cl^−^ to microbicidal HOCl [61]. An alternative view is that the role of the oxidase is to optimize the pH and ionic conditions within the vacuole for the killing and digestive functions of the neutral proteases [62], and that these conditions are entirely inappropriate for the peroxidatic activities of MPO [63]. It is important to note that the enzymology of MPO is complex and that it can function other than as a peroxidase, having superoxide dismutase [28] and catalatic [64] actions.

**Figure 3.**
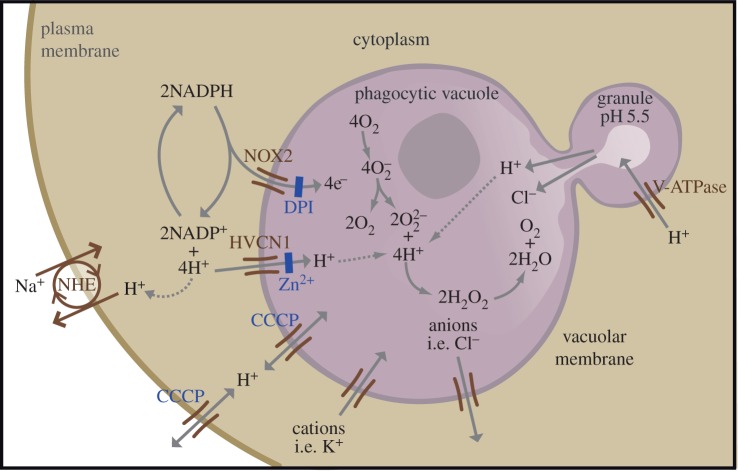
NOX2 in neutrophils, From [62]. NOX2 transfers electrons from NADPH in the cytosol onto O_2_ in the vacuole to produce 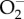 that dismutates to form 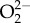 which is protonated to H_2_O_2_ that then breaks down to H_2_O and O_2_. The charge generated across the phagocytic vacuole is largely compensated not only by H^+^ passing into the vacuole through Hvcn1, but also by K^+^ passing in the same, and possibly Cl^−^ in the opposite, direction. The charge separation leaves H^+^ in the cytoplasm, which acidifies and protonation of 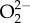 alkalinizes the vacuole. The alkaline vacuole and redistribution of ions activates the enzymes released from the granules that then kill and digest the microbe.

NOX2 passes electrons across the membrane of the phagocytic vacuole onto O_2_, to produce 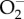 (figure 3). The transport of electrons into the phagocytic vacuole is electrogenic, causing a large, rapid membrane depolarization which will itself curtail further electron transport unless there is compensatory ion movement [65] by the passage of cations into the vacuole and/or anions in the opposite direction. The nature of the ions that compensate the charge will have a direct effect on the pH within the vacuole and the cytosol. The cytoplasmic granules are very acidic, with a pH of about 5.5, and they download their acid contents into the vacuole. The electrons that pass into the vacuole produce 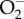 which dismutates to form peroxide (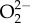) that is then protonated to form H_2_O_2_. The source of these protons will govern the alterations in the vacuolar pH. If all the charge is compensated by protons passing into the vacuole, then none of the protons released into the vacuole from the granules will be consumed, and the pH will remain acidic. In fact, most of the charge is compensated by protons passing from the cytoplasm into the vacuole through the HVCN1 channel, because if this channel is knocked out in mice the vacuolar pH becomes grossly elevated to about 11 (fig. 1 in [62]). Under normal physiological conditions, about 5–10% of the compensating charge is contributed by non-proton ions, some of which is K^+^ passing into the vacuole [66]; the residual ion flux could be the egress of chloride. These non-proton ion channels remain to be identified.

The plant response to infection and wounding shows remarkable similarities to the neutrophil oxidase. Damage to plants is followed by a response to isolate the exposed cells and tissues from the external environment by generating a protective barrier by the deposition of callose and polymerized phenolics, cell wall cross-linking and cell wall suberinization [67], which reduce water loss and protect against infection. The damage is rapidly followed by the influx of Ca^2+^ into the cytoplasm and damage-associated molecular pattern or pathogen associated molecular pattern receptor activation [68]. The binding of this Ca^2+^ to EF-hand motifs or phosphorylation [69] activates RbohD and RbohF [70,71] (and RbohC in roots [72]) to release 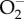 and K^+^ into the apoplastic space between the plasma membrane and the cell wall, the pH of which becomes elevated owing to the consumption of protons [73–75]. The apoplast contains superoxide dismutase [76], which produces H_2_O_2_ as substrate for apoplastic anionic peroxidase, a type III peroxidase [77] that cross-links substrates producing the suberization and lignification [78] to heal the wound. When the cells are damaged and normal metabolic homeostasis is disrupted, the normal cellular processes for regenerating reducing capacity will be impaired, and thus the antioxidant capability, which is dependent upon the constant reduction of molecules such as ascorbate and glutathione that are oxidized directly or indirectly by oxygen, is compromised. This allows the accumulation of H_2_O_2_ for the construction of the cross-linked barrier.

## 4. NOXs in the chemiosmotic generation of turgor

In filamentous fungi, filament growth, sporulation and penetration of the cuticle and cell wall of plants by germinating spores, and the opening of stomata by guard cells, extension of root hairs and growth of pollen tubes in plants are all processes that require the development of intracellular turgor. All these processes are also dependent upon the active participation of NOXs.

An examination of what we know of the neutrophil oxidase can explain how this turgor might be generated. The passage of electrons across the membrane depolarizes the membrane [65] and generates a charge that must be compensated to allow further electron transport. Whereas the electrons themselves, or charge compensating protons, do not have any osmotic consequences, other charge compensating ions do. In the neutrophil vacuole, the accumulation of cations, such as K^+^, in the phagocytic vacuole causes this compartment to swell [62]. In these cells, most of the charge is compensated by protons, so the ratio of osmotic effects to electron transport is much lower than would be the case if charge compensation was accomplished by other ions, as might be the case if the primary function of the oxidase were to increase tonicity. Although ion fluxes directly linked to NOXs at the plasma membrane of plants and fungi have not been measured directly, they must occur to compensate the charge generated across the plasma membrane by the electrogenic electron transport.

### 4.1. Fungi

Most fungi occur in the hyphae form as branching, threadlike tubular filaments. Genome sequencing of filamentous fungi showed early on that they have subfamilies of NOX enzymes sharing similarity to their mammalian homologues. NOX1 (NoxA) and NOX2 (NoxB) are homologues of the mammalian catalytic subunit gp91^phox^ (general review in [79]). The third NOX enzyme, NOX3 (NoxC), has been detected only in some fungi [80]. NOR1 (NoxR) is a regulatory subunit homologous to the mammalian p67^phox^. Another component of the NOX complex is a homologue of the small guanosine triphosphatase (GTPase) Rac1, which is required for pathogenicity and conidiogenesis in the rice fungal pathogen *Magnaporthe grisea* [81]. The tetraspanin PLS1 and a protein termed NoxD from *Botrytis cinerea* seem to be functionally and structurally related to the mammalian p22^phox^ [82], and Bem1 might represent a p40^phox^ analogue [83].

### 4.2. Fungi require NOXs to grow and infect

‘It thus appears that though turgor pressure is probably the driving force for hyphal extension, there is also an interplay between turgor pressure and wall biogenesis’ [84, p. 400]. The fungal mycelium consists of individual hyphae that radiate outwards from the colony centre. Each hypha grows solely by tip extension and generates new hyphae via the formation of lateral branches.

To achieve this characteristic pattern of mycelial organization, individual hyphae must exhibit apical dominance at the expense of other lateral branches [83]. The spitzenkörper is an intracellular organelle associated with tip growth. It is composed of an aggregation of membrane-bound vesicles containing cell wall components. The spitzenkörper is part of the endomembrane system of fungi, holding and releasing vesicles it receives from the Golgi apparatus. These vesicles travel to the cell membrane via the cytoskeleton and release their contents outside the cell by the process of exocytosis, where it can then be transported to where it is needed. Vesicle membranes contribute to growth of the cell membrane while their contents form new cell wall. The spitzenkörper moves along the apex of the hyphal strand and generates apical growth and branching; the apical growth rate of the hyphal strand parallels and is regulated by the movement of the spitzenkörper [85].

NOXs at the tip of the hyphae are required for apical dominance which several factors cooperate to achieve. There is a tip-high calcium gradient [86], which would activate NOXs through their EF hands [87] and NoxR, Rac1, Bem1 and Cdc42 are also important in this process [83,88].

For example, NOX1 homologues are required for fruiting body development [89], and NOX2 plays a key role in ascospore germination [90]. Ascomycetous fungi produce prodigious amounts of spores through both asexual and sexual reproduction. Their sexual spores (ascospores) develop within tubular sacs called asci that act as small water cannons, and expel the spores into the air, propelled by the osmotic pressure generated through the accumulation of KCl in the asci [91].

**Figure 4.**
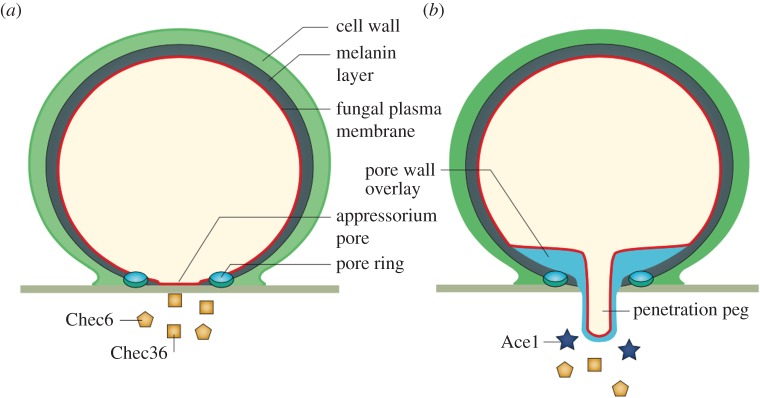
Mechanism of penetration of plant tissue by filamentous fungi. From [95]. (*a*) After attachment of the appressoria to the surface of the leaf at the pore ring, it secretes components onto the surface of the plant [100] through a pore with small apertures in the membrane. (*b*) Subsequently, this fenestration is closed off by a bilayer membrane that is continuous with the lining plasma membrane and the penetration peg. The structure is made impermeable to the passage of ions by the synthesis of a layer of melanin. Osmotic pressure then forces the penetration peg into the plant tissue.

NOXs at the tips of growing hyphae are ideally located for the development of appressoria. An appressorium is a flattened and thickened tip of a hyphal branch, formed by some parasitic fungi, that facilitates penetration of the host plant [92] or insect [93] cuticle. The germ tube is eventually shut off from the appressorium leaving the latter as a separate, independent unit that develops a spherical shape and becomes strongly attached to the plant cell by an adhesive ring of extracellular matrix. Anchored in this way a penetration peg is driven into the plant cell by osmotic pressure [94,95]. Experiments have been performed in which penetration pegs have been driven into inert synthetic non-biodegradable sheets, which suggested that pressures as high as 80 atmospheres (8 MPa) can be achieved [96]. To achieve these very high internal pressures, appressoria develop a lining of melanin that is permeable to water but impermeable to ions, thereby allowing the development of pressure through the internal accumulation of osmotically active molecules. NOXB produces hydrogen peroxide in a ring in the attachment zone between the appressorium and the leaf, which might be used to polymerize the ring of adhesive material [97] which has a very similar distribution and ring-like structure to that of H_2_O_2_ generation. The extracellular matrix that develops into the material that bonds the appressorium to the surface to which it attaches accumulates and fails to solidify when the appressorium is grown on a dialysis membrane [98], under which conditions the H_2_O_2_ would diffuse away. In the absence of NOXB [97] and/or NOXA [99], black spot disease and rice blast disease appressoria lose their infectivity and their penetration pegs, which would be in keeping with the loss of intracellular osmotic pressure owing to failure of the electrochemical power required to generate it (figure 4).

If the NOXs are responsible for generating the osmotic pressure within the appressorium from where would they obtain the osmotically active ions required to compensate the charge, given that the melanin lining makes the appressorium impermeable to ions? The answer could be that they are obtained from the invaded plant cell and that at least part of the purpose of the virulence factors secreted before peg development of the appressorium [95,100] is to permeabilize the plant cells to make these ions accessible. This does not fit with the fact that penetration pegs can be produced on polyester Mylar sheets [96]. The initial charge compensating ions could be contained within vesicles discharged into the space between the penetration peg and the target surface to initiate peg growth, which would then depend upon the uptake of ions from the penetrated tissue for further extension. In addition to the failure of robust bonding to the surface when the appressoria are grown on dialysis membranes as described above, these membranes prevent the production of penetration pegs, consistent with the requirement for extracellular diffusible factors for their development [98].

## 5. Plants

### 5.1. Pollen tubes

Sexual reproduction in flowering plants requires the male gamete, in the form of pollen, to reach the egg in the ovary. After landing on the stigma the pollen grain hydrates and germinates, during which a region of the pollen plasma membrane, the tip growth domain, is established and pollen tube elongation commences. This elongation in maize can occur at the prodigious rate of up to about 1 cm per hour and 1 ft in 24 h [101].

Growth is restricted to the tube tip where the cell wall must be deformable and requires the highly dynamic integration of new cell wall and membrane material, whereas the distal shank is more static and is required to resist turgor pressure. The tip membrane is replenished by vesicles that are transported by cytoskeletal elements and require the participation of GTPases and signalling molecules (reviewed in [101,102]). Extension also requires the active participation of two NOXs, RbohH and RbohJ [103], expressed at the growing tip, which are activated by high, micromolar, concentrations of Ca^2+^.

There are two main proposals for the mechanisms by which the pollen tube extension occurs [104]. The first is the cell wall model, in which it is proposed that the principal phenomenon responsible for the tube expansion is the exocytosis of vesicles containing pectins and other cell wall components such as cellulose, xyloglucans and callose that are either deposited by exocytosis or directly synthesized at the plasma membrane [105,106]. The alternative proposal is that the main driving force for growth is hydrodynamic pressure, which would also require the incorporation of newly synthesized plant wall material into the growing tip [104,107–109].

**Figure 5.**
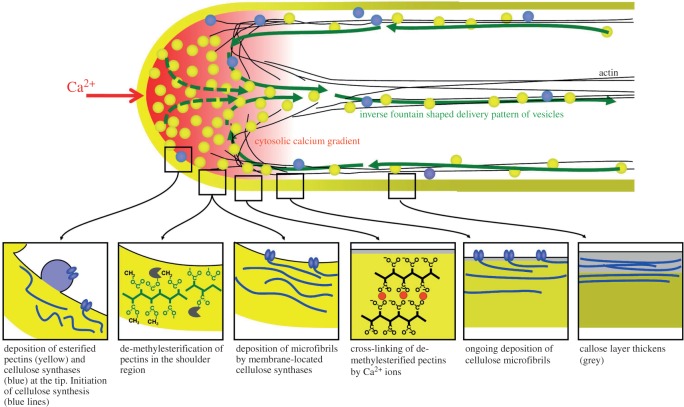
Spatial profile of mechanical properties and biochemical processes in the cell wall of growing pollen tubes. Reproduced from [110]. Mechanical modelling has shown that the precise spatial distribution of endomembrane trafficking, cell wall assembly processes and cross-linking of cell wall polymers determines the cellular morphogenesis and the resulting shape in growing plant cells. Rboh transfers electron from NADPH to O_2_ to form 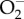. The charge produced across the plasma membrane must be compensated by the influx of anions, like Cl^−^, or the efflux of cations. Protons can also be exchanged for cations such as Na^+^ or K^+^. The influx of osmotically active ions would increase turgor and power tip growth.

Pollen tubes demonstrate fluxes of several ions in association with growth, which oscillates under artificial *in vitro* conditions [109]. To summarize the findings [110] (figure 5), it has been described that there is a zone of high Ca^2+^ just under the growing tip from which there is an efflux of chloride [111], a finding that has been disputed [112]. There is then an influx of chloride in the clear zone behind the tip where protons move out, leading to alkalinization, and K^+^ passes in. The pH just under the tip is acidic which has been attributed to an influx of protons. So how can the mechanisms underlying these findings be explained? NOXs are required for pollen tube growth and they are located precisely at the growing tip, and are activated by the influx of Ca^2+^. They expel electrons that form 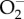. Chloride was measured with a vibrating-tip electrode, the specificity of which is dependent upon the selectivity of the chloride ionophore employed [111]. This does not appear have been tested against 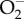, the measurement of which could have been misinterpreted as that of chloride in this region of the pollen tube. Rather than the description of an influx of protons to the region under the tip, an active oxidase would generate an acidic zone by virtue of the charge separation it produces, in which the reaction catalysed by NOX dictates the local release of protons from the oxidation of NADPH, as shown in figure 3. The other ion fluxes recorded could occur as a natural consequence of NOX activity. The chloride influx through SLAH3 [112] could compensate the charge induced by the expulsion of electrons. Cytosolic protons could contribute to the influx of extracellular K^+^, because low cytosolic pH regulates the gating of K^+^ channels; the low internal pH accelerated the activation kinetics of the K-uptake channel KAT1 expressed in *Xenopus* oocytes with a p*K*_a_ of 6 [113]. H^+^ extrusion through the plasma membrane ATPase [114] is also activated by low cytosolic pH and provides the electrogenic driving force for K^+^ uptake. The influx of osmotically active K^+^ and Cl^−^ into the cytoplasm would increase turgor to drive the tip forward.

### 5.2. Root hairs

Root hairs are tip-growing projections arising from single specialized root epidermal cells that markedly increase the surface area of the root. In *Arabidopsis*, a small bulge develops at the apical end of hair cells and this then elongates by polarized tip growth. The growth of root hairs has a lot in common with that of pollen tubes.

Root hairs are formed through tip growth [115,116], a process requiring synthesis of new cell wall material [117] and the precise targeting and integration of these components to a selected apical plasma membrane domain in the growing tips of these cells. The membranous material is transported in vesicles [118] by the actin cytoskeleton [119] under the regulation of small GTPases of the Rab, Arf and Rho/Rac families, along with their regulatory proteins, that are essential for spatio-temporal regulation of vesicular trafficking, cytoskeleton organization and signalling [117].

The *Arabidopsis thaliana* mutant *root hair defective 2* (*rhd2*) was shown to have a mutation in RbohC [120] and the maize *roothairless5* (*rth5*) in a monocot-specific NADPH oxidase rbohA [121]. One of the accessory proteins required for the function of the neutrophil NOX2 is Rac, a Rho GTPase that is complexed in the cytosol with a RhoGTPase GDP dissociation inhibitor [10]. Plants have homologues of RhoGTPases called ROPs. ROP2 [122] and a plant RhoGDI [123] are also required for 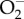 generation at the root hair tip, and for normal growth of root hairs. These results clearly demonstrate the requirement of plant NOX systems for normal root hair development.

RbohC accumulates just under the growing tip of root hair cells, in the same region that 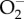 is generated, where it is then activated by the synergistic interaction of phosphorylation and an influx of Ca^2+^ that binds to its EF hands [124,125]. High, micromolar, concentrations of Ca^2+^ develop just under the extreme tip of the root hair [126,127], and there are indications that this Ca^2+^ influx is accomplished through Ca^2+^ channels that are opened by OH^−^ radicals. Ca^2+^ entering in this way would require it to move against the electrochemical gradient produced by electron efflux through RbohC and the resulting membrane depolarization. The oxidase produces an acidic region in the cytosol at the tip and one possibility that should be examined is that the electroneutral exchange of Ca^2+^ for protons might occur [128,129].

Measurements of pH at the surface of the growing root hair tip, and in the underlying cytosol, demonstrated reciprocal changes with the oxidase inducing an overlying alkalinization corresponding to cytosolic acidification [130]. Elevations in the surface pH and growth oscillated with the same periodicity but were out of phase. The application of ROS to rhd2 roots did not result in root hair growth [131], indicating that some other process associated with electron transport is likely to be responsible. The oxidase could drive tip extension by increasing turgor by moving osmotically active ions into the cytoplasm. Anions such as Cl^−^, 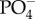 and 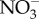 [132] would move down the electrochemical gradient generated across the plasma membrane by the passage of electrons. Cations could be exchanged for cytosolic protons through, for example, cation/H^+^ (CHX) exchangers [133] or pass in through K^+^ channels [134].

If the tip of the root hair is to advance through turgor pressure, then the shaft requires support to prevent expansion and rupture. This inelasticity is provided by the cell wall composed of cellulose, xyloglycan and pectin; shaft strength is compromised by defects in genes coding for the synthetic machinery [135,136], or for class III peroxidases [137] which are likely to cross-link the cell wall constituents. The H_2_O_2_ substrate for these peroxidases could come from dismutation of the 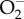 generated at the tip.

### 5.3. Guard cells

Stomatal opening is the most obvious process in plants in which changes in turgor play an essential role and in which NOXs, in this case AtrbohF and /or AtrbohD, are required, not to increase turgor but to decrease it. Stomata are microscopic pores in the epidermis of aerial plant parts that through which oxygen and water vapour are lost and CO_2_ enters for photosynthesis. Stomatal opening and closing is regulated by the reversible swelling and shrinking of a pair of guard cells that surround the aperture. Opening occurs when the turgor pressure is increased by the uptake and intracellular generation of solutes, and the osmotic pressure that they generate, which causes an outward swelling of the guard cells that separate, thereby increasing the aperture. During stomatal closure, there is a reversal of the process with the loss of solutes and water from the guard cells, with a consequent deflation and aperture narrowing. Guard cell volumes can vary by as much as 40% during this cycle [138].

All solute uptake and efflux must occur through ion channels (down an energetic gradient) or transporters (requiring energy, generally in the form of ATP) in the plasma membrane. Rates of fluxes through ion channels are orders of magnitude greater than those through transporters.

A large number of such channels and transporters are involved in the regulation of the stomatal aperture (reviewed in [139]). I am presenting here a highly simplified scheme that takes account of molecules that have been shown to alter stomatal opening and closing when their genes are targeted.

Guard cells contain a large vacuole that accounts for a significant proportion of the cell volume and parallel changes in its volume are integral to the swelling and shrinking cycle. The membrane that bounds this vacuole is called the tonoplast.

**Figure 6.**
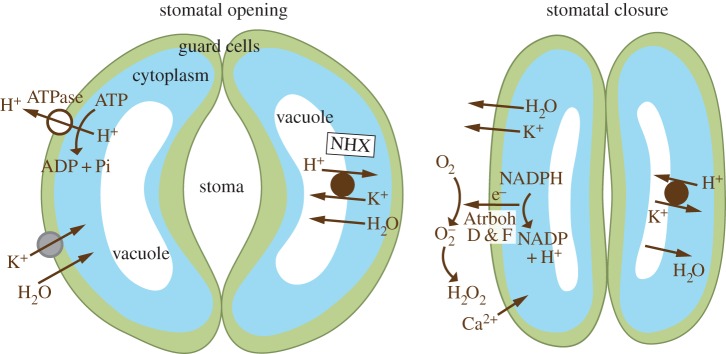
Opening and closing of stomata by guard cells. A highly simplified scheme (adapted from [139]) of a mechanism by which guard cell turgor could vary stomatal opening and closing by involving Na^+^,K^+^/H^+^ antiporters (NHX) [140] and AtrbohD and F NOXs [141], all of which impair guard cell fluctuations in volume if their genes are knocked out.

A simplified scheme of the mechanisms regulating the changes in guard cell volume is shown in figure 6. It largely centres around a cycle of fluxes of H^+^ in exchange for K^+^ and H_2_O. H^+^ accumulates in the vacuole as shrinkage occurs and is then expelled, first from the vacuole and then from the cell in exchange for K^+^ and H_2_O, which causes the cell to swell [142].

As might be expected, the influx of CO_2_ for photosynthesis is activated by stomatal opening when exposed to light. This induces a hyperpolarization of the plasma membrane by activating H^+^ extrusion through a plasma membrane ATPase [143,144]. K^+^ enters down the electrochemical gradient through a variety of inward rectifying channels [145] taking water with it. K^+^ then enters and induces swelling in the vacuole in exchange for H^+^ through Na^+^, K^+^/H^+^ (NHX) antiporters in the tonoplast [140].

Stomatal closure as a consequence of guard cell shrinkage is a reversal of these ion fluxes. The initial event is depolarization of the plasma membrane that follows Ca^2+^ oscillations [146] which inactivate the inwardly rectifying K^+^ channels. The depolarization is thought to occur through the loss of anions such as Cl^−^, malate^2−^ and 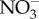 through R- and S-type anion channels [147], but these channels are opened by membrane depolarization [148], so what is the driving force for this to occur? Guard cells are enriched in the NOXs AtrbohD and AtrbohF, and stomatal closure is compromised in the absence of these molecules [149], which can perform the essential functions required for loss of guard cell turgor. Plasma membrane NOXs are ideally suited to generate the driving force for outwardly rectifying voltage-gated K^+^ channels [150] through electron-transport-mediated membrane depolarization. They will be activated by increased cytosolic Ca^2+^, the transfer of electrons to the exterior will depolarize the membrane, which will drive charge compensating K^+^ to the exterior through voltage-gated K^+^ channels, together with the release of anions through depolarization-responsive R- and S-type channels. H_2_O_2_ generation at the plasma membrane activates Ca^2+^ influx [151] which could amplify 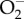 generation. In addition, the vacuole must re-establish its pool of H^+^ ions to be in a position to exchange these for K^+^ when next required to swell, and the H^+^ originally expelled through the plasma membrane ATPase must be regenerated. The H_2_O_2_-induced Ca^2+^ influx could also activate K^+^/H^+^ exchange through NHX exchangers [152]. The regeneration of cytoplasmic H^+^ from NADPH when this is converted to NADP^+^ will accomplish this end. The fact that the cytoplasmic pH rises as guard cells shrink is not incompatible with the generation of H^+^ in the cytosol by the Atrbohs, because the pH is a reflection of the relative rates of uptake of H^+^ into the vacuole as compared with generation in the cytosol, and will be alkaline if vacuolar uptake predominates.

## 6. Blood vessel endothelial cells

‘The vascular endothelium represents a population of squamous epithelial cells characterized by a particular histological localization (intima of blood vessels) and by several physiological functions such as the transport of substances between blood and tissues, the modulation of the vascular tone, the control of blood coagulation and that of the leucocyte extravasation. In spite of all these elements in common and of an identical embryonic origin, endothelial cells show definite morphological and physiological variations that divide them into types and subtypes, each specifically associated with various categories of organs. Even within the vasculature of the same organ, there are clear segmental (arterial/capillary/venous) differentiations of the endothelial cells. While the morphological and physiological differences between endothelial cells are well documented, there are very few data on the biochemistry underlying this heterogeneity. The luminal aspect of the endothelial plasmalemma is a compartment of crucial importance in the biology and pathology of the cardiovascular system’ [153 p. 381, 154].

Given the clear description of the ion fluxes and pH changes induced by NOX2 in neutrophils, and on the balance of probability the changes in turgor produced in fungi and plant cells by NOX-induced ion fluxes, it is interesting to speculate on the role of NOXs in the vasculature, about which much less is known. NOX-stimulated fluctuations in the passage of ions and accompanying H_2_O between endothelial cells and blood plasma could have important influences on blood pressure and tissue perfusion because of the major influence that even minor changes in the radius of the vessel lumen have on flow. According to Poiseuille's law, in the case of smooth flow (laminar flow), the volume flowrate is given by the pressure difference divided by the viscous resistance, and whereas there is a linear relationship between resistance, viscosity and length, resistance is related to the fourth power of the radius.

Although the role of the microvasculature is not clear [155], the regulation of blood pressure has largely been attributed to smooth muscle tone in the precapillary arterioles [156]. Vasoactive signals originating in capillaries can govern capillary blood flow, and the endothelium functions as a highly effective pathway for the conduction of electrical signals in the microcirculation [157]. Because they are not surrounded by smooth muscle cells, the role of capillaries in regulating their own perfusion has largely been ignored, and it is assumed that they signal to the arterioles from which they originate. It is, however, possible that capillaries can also control their own perfusion by regulating the volume of their endothelial cells, and through that their radius.

There is an extensive literature on NOXs in endothelial cells (reviewed in [158–160]), which express four NOXs: NOX1, NOX2, NOX4 and NOX5. The architecture of the vascular tree is complex, and very different depending upon whether it is in the arterial, venous or lymphatic system, and upon the size of the vessels, and it is intimately connected to other organs such as the nervous system. NOXs might play a similar role in lymphatic capillary endothelial cells [161], but there is no literature on this subject.

### 6.1. Subcellular distribution

The subcellular distribution of the NOXs within the endothelial cells is absolutely critical to their function. Given that all other NOXs appear to be located in plasma membranes, and confluent cultures of primary bovine [162,163] and human [164] endothelial cells secrete 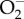 from their luminal surfaces into the extracellular medium, as evidenced by the reduction of cytochrome *c*, which does not penetrate cells, reports of intracellular [165–167] locations of NOXs in endothelial cells merit examination.

The most comprehensive of these [165,167] reported that the subcellular location of the endothelial gp91-^phox^ and p22-^phox^ subunits was significantly different from that reported for the neutrophil oxidase, in that they were predominantly intracellular and colocated in the vicinity of the endoplasmic reticulum. These data must be interpreted in the light of the experimental techniques employed. The localization by immunohistochemistry was performed on bovine and porcine endothelial cells with antibodies made against human proteins. The subcellular fractionation was by differential rather than isopyknic centrifugation, which is suboptimal. The cells were frozen and thawed and extensively sonicated before use, which can solubilize organelles, and the marker enzymes used to determine the distribution of the organelles were expressed as specific (as a function of the protein concentration) rather than as total activities, which can be misleading. Another publication [166] reported that NOX2, p22^phox^ and p47^phox^ are targeted to the nuclear pore complex. They used H9c2 cells, a rat cardiomyoblast cell line, and once again, the antibodies used in the immunohistochemistry appear to have been raised against the human proteins. Finally, studies were conducted on EaHy926 cells [168], described as an endothelial cell line. In fact, these cells are a somatic cell hybrid in which human umbilical vascular endothelial cells were fused with a human lung carcinoma cell line [169]. The results shown for these cells just as well reflect the properties of the carcinoma cells as the endothelial cells. Commercial antibodies were used, which will have been subject to all the vagaries of these products. Considering the large body of evidence from plants and animals that the NOX system is found in the plasma membrane, the contradictory findings provided for alternative locations in endothelial cells do not appear to be of sufficient weight to overturn the general paradigm of a plasma membrane location for these electron transport chains.

### 6.2. NOXs and the regulation of blood pressure

It is generally accepted in the literature that 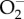 produced by endothelial cell NOXs regulates the vasodilatory action of NO by combining with it to produce peroxynitrite. It was initially shown, in an organ bath system in which the effluent from endothelial cells cultured on beads was perfused over aortic smooth muscle strips, that endothelium-derived relaxation factor (EDRF) was prevented from breakdown by superoxide dismutase, suggesting that 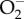 normally degrades EDRF, subsequently identified as NO [170]. Cu^2+^ had a more prolonged effect than superoxide dismutase although no dismutase activity of the Cu^2+^ was demonstrated [171].

It was demonstrated *in vitro* that NO reacted rapidly with 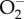 [172,173] to generate peroxynitrite [172], from which it was surmised that these reactions take place *in vivo*. There is now a very large literature on peroxynitrite and its possible roles in the cardiovascular system (reviewed in [174]) with very little direct evidence to this effect.

**Figure 7.**
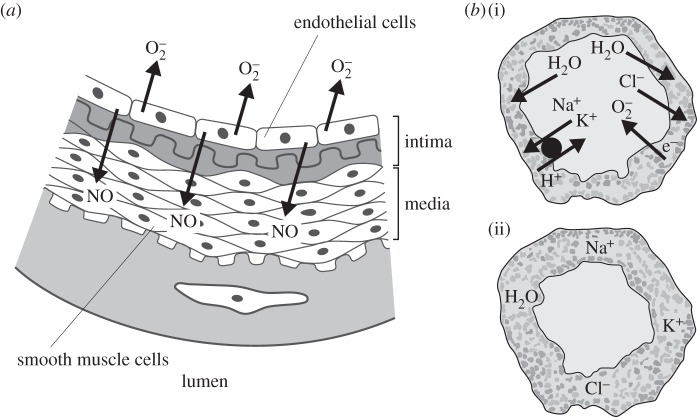
Mechanisms by which blood vessels might regulate blood pressure. (*a*) It is generally accepted that endothelial cells generate 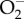 and NO which react to produce peroxynitrite and that by consuming NO the 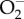 limits the vasodilatation produced by the relaxation of the smooth muscle cells by NO. However, the NO and 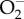 are released in different directions, the NO towards the smooth muscle cells in the media and 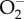 into the lumen. (*b*) A scheme by which capillaries could vary the diameter of their lumen. NOX activity could drive osmotically active ions in (*b*(ii)) or out of the endothelial cell cytoplasm. The nature of the charge compensating ions and the consequent swelling or shrinkage of the cell would depend upon the characteristics of the voltage-gated channels.

It is essential to know the topographical distribution and function of the NOXs and NO synthases. The initial description of the degradation of NO by 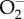 used an organ bath in which the natural segregation of 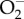 and NO generation were artificially disrupted. The primary effect of NO on the vascular smooth muscle cells must indicate release of NO from the basal side of the endothelium, whereas the measurement of 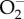 release from the luminal surface, as measured by reduction on the non-penetrating cytochrome *c*, suggests that 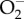 and NO are released in opposite directions and that they are unlikely to interact in a major, controlled way (figure 7). In addition, little is known of the relative stoichiometry of the generation of these compounds.

### 6.3. Animal models

NOXs 1, 2 and 4 and their associated molecules have been knocked-out or over-expressed in mice, and the outcomes of their effects on blood pressure and vascularization assessed after a variety of stimuli and insults [158]. Most of the studies on knock-out mice were performed on heterozygote animals and clear trends were not forthcoming.

Unfortunately, the distribution of the different NOXs in the different blood vessels and organ beds has not been determined, which makes the assessment of the effects of knocking-out or over-expressing any one of the three NOXs very much a hit or miss affair.

Basal blood pressure is abnormally low in NOX1^−/y^ [175], and NOX2^−/y^ [176,177] mice, and NOX4^−/−^ Dahl salt-sensitive rats demonstrate a reduction in salt-induced hypertension [178]. Renal blood flow is higher and renal vascular resistance lower in NOX2^−/y^ mice. In p47^−/−^ mice, there was marked blunting of the hypertensive effect of the infusion of angiotensin II [179].

If NOXs do prove to be important in the regulation of blood pressure, then the production of selective modulators could be very effective in its regulation [180].

### 6.4. NOX2 deficient humans with chronic granulomatous disease

There does appear to be an abnormality of the vasculature in patients with a defective NOX2 system resulting in CGD. In a study involving 17 patients with X-linked (NOX2^−/y^) or autosomal (p47^phox−/−^) CGD, although not compared statistically, the overall diameter of the brachial arteries in the patients was less than that of controls [181]. In another investigation, the internal carotid artery wall volume was found to be significantly (22%) lower in the 41 CGD patients than in the 25 controls [182]. Whether this is a direct effect on the large vessels, or an adaptation to alterations in peripheral resistance, or perfusion, as a result of changes to the capillary bed remains to be established.

## 7. Conclusion

The optimal design of the NOX electron transport chain has resulted in its remaining largely unchanged during evolution from red algae, fungi and plants to man. It provides the simplest means by which electrons can be efficiently passed across membranes, using an abundant and metabolically efficient substrate, a ubiquitous redox cofactor, two haems, one on either side of the membrane, and the commonest electron acceptor, oxygen. The potential energy produced by the separation of charge across the membrane is then used in various applications, depending upon the nature and characteristics of the voltage-gated ion channels and exchangers that are present and activated in the different organisms and tissues. The commonest use of this electrochemical energy in nature appears to be in the development of osmotic turgor required for the growth of tubular structures in fungi and plants, or the cyclical opening and closing of stomata in the latter. In addition to pumping ions, in neutrophils, NOX2 functions to alter pH in the phagocytic vacuole and NOX3 might have a similar function in the inner ear [183]. The role of the NOXs in endothelial cells of blood vessels and their regulatory mechanisms remain to be discovered, but they could be important in the control of blood pressure and tissue perfusion.
